# Antimicrobial Potential of Nanomaterials Synthesized with Extracts from *Annona* Plants: A Review

**DOI:** 10.3390/antibiotics14080748

**Published:** 2025-07-24

**Authors:** Yared Gutiérrez-Pinzón, Alma Hortensia Martínez-Preciado, José Miguel Velázquez-López, Cristina Pech-Jiménez, Víctor Manuel Zúñiga-Mayo, Santiago José Guevara-Martínez, Gilberto Velázquez-Juárez

**Affiliations:** 1 Department of Chemical Engineering, University Center of Exact Sciences and Engineering, University of Guadalajara, Blvd. Marcelino García Barragán 1421, Guadalajara 44430, Jalisco, Mexico; yared.gutierrez8804@alumnos.udg.mx (Y.G.-P.); alma.martinez@academicos.udg.mx (A.H.M.-P.); 2 Department of Chemistry, University Center of Exact Sciences and Engineering, University of Guadalajara, Blvd. Marcelino García Barragán 1421, Guadalajara 44430, Jalisco, Mexico; jmiguel.velazquez@academicos.udg.mx; 3 Department of Human Reproduction, Growth and Child Development, University Center of Health Sciences, University of Guadalajara, Guadalajara 44350, Jalisco, Mexico; elma.pech3708@alumnos.udg.mx; 4 CONACyT–Institute of Phytosanitary Research, Colegio de Postgraduados, Montecillo Campus, Texcoco 56230, State of Mexico, Mexico; zuniga.victor@colpos.mx; 5 Department of Pharmacobiology, University Center of Exact Sciences and Engineering, University of Guadalajara, Blvd. Marcelino García Barragán 1421, Guadalajara 44430, Jalisco, Mexico; santiago.guevara@academicos.udg.mx

**Keywords:** *Annona*, acetogenin, phytochemical, green synthesis, phytosynthesis, nanomaterial, antimicrobial, nanoparticles

## Abstract

Plants of the *Annona* genus have garnered increasing scientific interest due to their rich phytochemical profile and broad spectrum of biological activities, which include antimicrobial, antiproliferative, and cytotoxic effects. Among the most studied compounds are acetogenins and *Annona*cins, which exhibit potent bioactivity and have been identified as key agents in the green synthesis and stabilization of nanomaterials. In recent years, the integration of *Annona* plant extracts—particularly from leaves—into nanotechnology platforms has opened new avenues in the development of eco-friendly and biocompatible nanostructures for biomedical applications. This review provides a comprehensive overview of the current knowledge regarding the antimicrobial properties of nanomaterials synthesized using extracts from *Annona* species. This review encompasses 74 indexed articles published between 2012 and 2023, focusing on the synthesis of nanomaterials using extracts from this genus that exhibit antimicrobial and biomedical properties. The search was conducted in databases such as Google Scholar, Web of Science, and Scopus. Emphasis is placed on their antibacterial, antifungal, and anthelmintic effects, as well as additional therapeutic potentials, such as antidiabetic, antihypertensive, antiproliferative, and cytotoxic activities. The analysis of the recent literature highlights how *Annona*-derived phytochemicals contribute significantly to the functionalization and enhanced biological performance of these nanomaterials. This work aims to support future research focused on the rational design of *Annona*-based nanostructures as promising candidates in antimicrobial and therapeutic strategies.

## 1. Introduction

The alarming rise of antibiotic-resistant pathogenic bacteria has intensified the global search for effective, safe, and biocompatible antimicrobial agents [1,2]. In parallel, novel materials have been developed to deliver drugs or bioactive compounds to targeted cells. Among these, nanomaterials have gained increasing attention due to their unique properties and ease of production.

Nanomaterials are defined as materials with at least one dimension smaller than 100 nm, a scale that imparts them with distinctive physical and chemical properties [3,4]. Generally, two primary approaches exist for producing nanomaterials: the top-down method, which involves breaking bulk materials into nanoparticles using techniques such as laser ablation, grinding, and milling [5], and the bottom-up method, which assembles nanoparticles from atomic or molecular precursors using chemical reducing agents such as sodium borohydride, 1,2-hexadecanediol, or sodium citrate. However, these conventional methods are usually environmentally hazardous, expensive, and require highly trained personnel.

To overcome these drawbacks, green synthesis methods have emerged as an eco-friendly and sustainable alternative. Since the early 2000s, the use of biological systems including plant extracts, microbial cultures, and fungal biomass-has become common in the biosynthesis of metal nanoparticles [6]. This approach, often termed “green synthesis” or “phytosynthesis” when using plant extracts [7], has been recognized for its simplicity, low cost, environmental sustainability, and biocompatibility with eukaryotic cells [8,9]. A key factor in this process is the presence of secondary metabolites, such as alkaloids, terpenoids, tannins, flavonoids, phenols, quinones, steroids, carbohydrates, lipids, proteins, amino acids, and enzymes, which possess antioxidant activity and act as natural reducing and capping agents. It has been demonstrated that these metabolites have the capacity to stabilise nanoparticles, thereby enhancing their therapeutic potential, particularly in the context of antimicrobial applications.

Plant extracts have shown remarkable potential for the green synthesis of nanomaterials. Commonly, plant extracts act simultaneously as reducing and stabilizing agents due to the rich content of flavonoids, alkaloids, terpenoids, and phenolic compounds [8] Furthermore, plant-derived nanomaterials often exhibit intrinsic antimicrobial properties, enhancing their utility in combating multidrug-resistant microorganisms. In this context, species of the *Annona* genus (family *Annona*ceae) have gained scientific interest due to their wide range of bioactive phytochemicals, particularly acetogenins, which are known for their antimicrobial, cytotoxic, and anti-inflammatory effects. Recent studies have explored the green synthesis of nanomaterials using extracts from *Annona*, leveraging both the intrinsic bioactivity of their compounds and the functional properties of nanoparticles [10]. Thus, nanomaterials synthesized with *Annona* extracts represent a promising strategy for developing next-generation antimicrobial agents.

The *Annona*ceae family is one of the most diverse of the angiosperms, comprising over 130 genera and approximately 2300 species. Among them, the genus *Annona* is the most prolific with 119 species [11]. Traditionally, Anonna fruits and leaves have been used in folk medicine to treat conditions such as cancer, diabetes, hypertension, and gastrointestinal and skin infections [4,12,13,14,15,16,17,18]. The pharmacological potential of *Annona* species is attributed to a complex phytochemical profile that includes not only secondary metabolites but also proteins, peptides, sugars, and lipids [15,16,17,19]. Notably, *Annona* extracts contain compounds exclusive to the genus that exhibit antiparasitic, antioxidant, antidiabetic, anticancer, antiproliferative, and antimicrobial activities [12,13,15,17,20].

Despite increasing interest in the bioactivity of *Annona* species, there is a lack of comprehensive reviews that explore their role in nanotechnology, particularly in the green synthesis of antimicrobial nanomaterials. Existing reviews often focus on the pharmacological properties of *Annona* phytochemicals or general aspects of nanoparticle biosynthesis, but not on their intersection [15,21,22,23]. This review aims to fill the gap by systematically examining the use of *Annona* plant extracts in the synthesis of nanomaterials and highlighting their antimicrobial potential. We discuss the unique advantages of *Annona* species over other plant sources—such as their exclusive bioactive compounds, availability, and versatility in nanoparticle synthesis—and evaluate how these characteristics contribute to the development of efficient, eco-friendly nanomaterials for biomedical applications.

This review focuses on the applications of secondary metabolite-derived nanomaterials produced from extracts of plants in the genus *Annona*, which contain exclusive compounds with antimicrobial activity that can be widely utilized in biomedicine. Plant extracts from the *Annona* genus have emerged as an alternative for the synthesis of nanoparticles due to their rich phytochemical composition, including acetogenins, alkaloids, flavonoids, and phenolic compounds, which act as reducing and stabilizing agents, promoting the formation of nanostructures. These molecules also enhance the bioactivity of the resulting nanomaterial, giving it antimicrobial, antioxidant, anticancer, and cytotoxic properties [18,20,24,25,26]. In addition, several species of *Annona* have demonstrated remarkable versatility in synthesizing metallic and polymeric nanoparticles, which, combined with their availability and low cost, make them a highly efficient and environmentally friendly source for nanobiotechnology. Although there are some reviews on the use of *Annona*, these have focused on explaining the versatile attributes of the phytochemicals of the different species of the genus, but not their application in conjunction with nanomaterials.

## 2. Phytochemical Composition of Annona Extracts

### 2.1. Acetogenins

Plants of the genus *Annona* produce a wide array of bioactive secondary metabolites with therapeutic potential. These include acetogenins, alkaloids, flavonoids, phenols, tannins, and terpenoids, as well as biomolecules such as lipids, proteins, polysaccharides, and cyclo-oligopeptides, many of which contribute to antioxidant, cytotoxic, and antimicrobial activities [15,27].

Acetogenins are unique to the *Annona* genus and are among its most studied metabolites [28]. Structurally, they are long-chain lipophilic peptides (C35–C37), typically containing one to three tetrahydrofuran (THF) rings, as well as the presence of other functional groups such as hydroxyls, ketones, and epoxides. They are usually characterized by a combination of fatty acids with a 2-propanol unit at C-2 that forms a methyl-substituted α,β-unsaturated γ-lactone [29]. More than 500 different acetogenins have been identified [30]. Several studies have reported on the antimicrobial and antifungal activity of the acetogenin-rich fraction. For example, Paula-Terezan et al. [31] obtained *A. coriacea* leaf extracts, demonstrating antimicrobial activity against *Streptococcus mutans*, *S. mitis*, *S. sanguinis*, and *S. salivarius*, while Aguilar-Hernández et al. [32] purified acetogenins from the endosperm of *A. muricata* seeds, demonstrating antimicrobial activity against *Enterococcus faecalis*, *Listeria monocytogenes*, *Aeromonas hydrophila*, *Burkholderia cenocepacia*, and *Salmonella paratyphi*. Additionally, acetogenins isolated from *A. muricata* seeds have shown antifungal activity against *Candida albicans*, *Candida krusei*, and *Candida tropicalis*, as reported by [32]. Their bioactivity has been attributed to inhibition of mitochondrial complex I, leading to ATP depletion and apoptosis. Additionally, their THF groups may chelate divalent cations (e.g., Ca^2+^, Mg^2+^), disrupting ionic homeostasis and membrane potential [29]. Specific acetogenins with antimicrobial activity have been identified. For instance, squamocin G is a potent acetogenin isolated from dichloromethane extracts of *A. glabra*, *A. muricata*, and *A. squamosa* that has shown significant activity against *Colletotrichum* sp. and *Fusarium* sp. [33].

Due to their hydrophobicity, acetogenins exhibit low solubility in aqueous solutions, which limits their biomedical applications [34]. To address this, nanoformulations such as nanoemulsions and liposomes [35,36] have been utilized to enhance solubility, stability, and bioavailability, thereby improving their efficacy in both in vitro and in vivo models.

### 2.2. Phenolic Compounds

Phenolic compounds possess antioxidant activity due to multiple hydroxyl groups capable of electron donation [37]. These include simple phenolic acids, flavonoids, stilbenes, and tannins, which vary depending on plant tissue, environmental conditions, and extraction methods [38]. Phenolics are widely recognized as reducing agents in green synthesis, facilitating metal ion reduction (e.g., Ag^+^ to Ag^0^) and acting as capping agents by adsorbing onto nanoparticle surfaces, preventing aggregation [39,40]. Among the compounds reported to have been extracted from *Annona* tissue preparations are derivatives of cinnamic acid and p-coumaric acid, gallic acid, catechin, and epigallocatechin [41]. Although most studies focus on selectively extracting fractions with solvents and then evaluating their antimicrobial potential, some studies have already succeeded in identifying specific phenolic molecules with antimicrobial properties.

Polyphenolic extracts from *Annona* species, particularly *A. muricata* and *A. cherimola*, have demonstrated high concentrations of bioactive compounds and notable antimicrobial activity. Nolasco-Gonzalez et al. [42] reported that ultrasound-assisted extraction from *A. muricata* leaves produced extracts that exhibited antibacterial effects against both Gram-positive and Gram-negative bacteria, with no toxicity observed in *Artemia salina* bioassays, supporting their biocompatibility and therapeutic potential. Similarly, Aguilar-Villalba et al. [12] demonstrated that phenolic-rich extracts from *A. cherimola* displayed significant antibacterial activity against *S. aureus*, attributed to oxidative stress induction and cell membrane disruption. While polyphenols such as p-coumaric acid, gallic acid, and catechins are widely recognized for their antimicrobial action in other plant systems, their abundance in *Annona* species [43], often accompanied by synergistic phytochemicals like acetogenins, amplifies their effects and enhances their role in green nanoparticle synthesis.

### 2.3. Alkaloids and Terpenoids

Alkaloids are nitrogen-containing compounds with diverse structures (isoquinoline, pyridine, indole, pyrrole) and are typically extracted from leaves, seeds, fruits, and peels [44]. They exhibit antioxidant, antimicrobial, anti-inflammatory, antidiabetic, and cytotoxic effects [45]. Alkaloids can participate in nanoparticle synthesis by donating electrons during redox reactions and by coordinating metal ions, thus stabilizing nanostructures [46]. Species of *Annona* vary considerably in their alkaloid content, which appears to influence their antimicrobial potential [47]. For instance, *A. squamosa* contains alkaloids such as anonaine, asimilobine, corypalmine, liriodenine, nornuciferine, and reticuline in both leaves and seeds. Extracts from these species have shown minimum inhibitory concentration ranging from 39 to 78 ug/mL against *S. aureus*, *K. pneumoniae*, and *E. faecalis* [48]. Likewise, *A. purpurea* has been found to contain more than 30 distinct alkaloids, including annomontine and oxopurpurereine, with antifungal activity that varies seasonally in correlation with alkaloid abundance [49].

Terpenoids, composed of isoprene units, have demonstrated antimicrobial, antioxidant, and anti-inflammatory activities [50]. Terpene profiles differ among *Annona* species. For example, essential oils from *A. cherimola*, *A. squamosa*, *A. muricata*, and *A. glabra* include high levels of β-elemene (25.02%), β-caryophyllene (37.11%), bicycloelemene (23.58%), and β-gurjunene (42.49%) [51]. Additionally, *A. senegalensis* produces kaurenoic acid, a diterpene with reported antibacterial activity at minimum inhibitory concentrations of 30–180 µg/mL [52]. Studies have also identified isoquinoline alkaloids, sesquiterpene lactones, and various other terpenoids in *Annona* extracts [44,53,54], which have shown inhibitory effects against both common and multidrug-resistant bacteria, as well as diverse fungal species. Although differences in extraction protocols and testing methods must be considered, variations in metabolite composition generally align with differences in antimicrobial efficacy across *Annona* species. Among terpenoids, there is an important subfamily called phytosterols, mainly isolated from *Annona* seeds, which are structurally similar to cholesterol and have shown antiproliferative activity in acute myeloid leukemia cell lines [55]. Both alkaloids and terpenes can coordinate metal ions and act as stabilizing ligands. In particular, some terpenes with double bonds or reactive functional groups can participate in redox reactions and in controlling the size and shape of nanoparticles [56,57].

### 2.4. Other Metabolites

Studies report that *Annona* species yield additional compound classes beyond terpenoids, terpenes, alkaloids, acetogenins, and phenolics. In particular, steroids were isolated from *Annona cherimolia* (seeds), *Annona glabra* (fruits and stems), and *Annona pickelii* (stem bark). Glycosides were found in *Annona cherimolia* seeds, and *Annona* glabra provided amides (N-trans-feruloyltyramine and N-p-coumaroyltyramine) together with a nitrogen-containing azaanthraquinone derivative [21,27,58,59].

**Figure 1:**
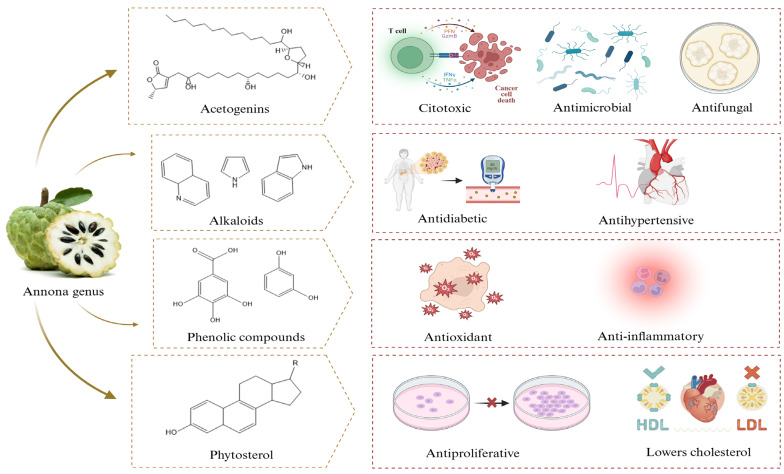
Biological activity of phytochemicals from the *Annona* genus, supporting traditional and potential therapeutic applications of *Annona* extracts in various biomedical fields.

Figure 1 provides a synopsis of the biological activity exhibited by the phytochemicals present in the extracts of the *Annona* genus.

## 3. Phytosynthesis of Nanomaterials with Annona Extracts

**Figure 2:**
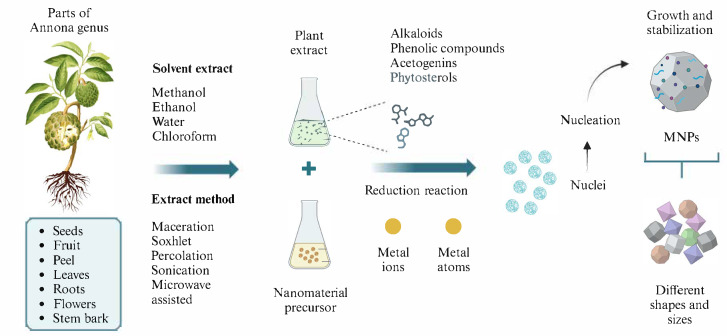
General methodology for the Phytosynthesis of metal nanoparticles using *Annona* extracts. Biosynthesis of nanomaterials allows reduction, nucleation, and stabilization reactions to occur in a single step, guided by the phytochemicals present in the plant extracts. The choice of solvents and extraction methods must be considered in the green synthesis of nanomaterials from plant extracts.

Green synthesis, also known as phytosynthesis, is a sustainable and eco-friendly strategy for fabricating nanomaterials, particularly metal-based nanoparticles. This approach condenses three essential steps in nanoparticle production—metal ion reduction, nucleation, and stabilization—into a single process mediated by plant-derived extracts [1,2,39,46]. Extracts from *Annona* species are notably rich in phytochemicals, which actively participate in these stages by donating electrons, coordinating metal ions, and capping nanoparticle surfaces [60,61,62]. Figure 2 illustrates the phytosynthetic route for nanoparticle formation using *Annona* extracts.

Several studies have reported the synthesis of nanoparticles using extracts from *Annona*. However, the wide variability in experimental conditions—including the type of metal, particle shape, solvent, *Annona* species, and plant tissue used—makes it challenging to analyze and compare the data comprehensively. To identify current trends, this review analyzed publications from 2012 to 2023 focused on the synthesis of nanoparticles using *Annona*-derived extracts. The keywords “*Annona*” and “nano” were combined using the Boolean operator “AND” to search databases such as Web of Science, Google Scholar, and Scopus. Only articles reporting biomedical applications were included; those lacking such context were excluded.

A total of 74 articles were included, of which 60.53% are studies based on the synthesis of nanoparticles using extracts of the *Annona muricata* species: 27.63% of *Annona squamosa*, 7.89% of *Annona reticulata*, 2.63% of *Annona glabra*, and 1.32% of *Annona diversifolia*. Among the synthesized nanomaterials, silver nanoparticles (AgNPs) were the most frequently reported (48.64%), followed by copper nanoparticles (8.10%), chitosan (8.10%), selenium nanoparticles (5.4%), and gold nanoparticles (4%). In general, there has been a preference for the green synthesis of silver nanomaterials due to their versatility in the synthesis process. Since it requires conditions that do not demand greater maintenance or specialized equipment, such as a neutral pH, it is usually carried out in aqueous solvents. Additionally, silver nanomaterials exhibit a wide range of biological activities, including antimicrobial and antifungal properties [39,40]. In contrast to other metals such as gold, platinum, or zinc, silver is more economical and accessible, and its colloidal chemistry is well known, facilitating its handling and characterization. All of this has contributed to its predominant use in green synthesis studies, particularly with extracts from plants of the *Annona* genus.

With a rating of 62.43%, the preferred method of nanomaterial synthesis was stirring, followed by heating with stirring, with a preference rating of 14.29%. Ion gelation was the next most preferred method, with a rating of 10.39%. The least used methods were the use of UV lamps and microwaves. The stirring method is more efficient in homogenizing the reactants and promoting controlled nucleation. This method is particularly popular in synthesis with plant extracts because it requires the phytochemicals present in the plant extracts to be dispersed, allowing them to react effectively with the metal precursors, which is more easily achieved through continuous stirring. Additionally, moderate heating accelerates reduction reactions, enhances the colloidal stability of the nanoparticles formed, and promotes the formation of smaller and more uniform sizes. In contrast, the drip method, although helpful in controlling the addition rate of the precursor, can limit the homogeneity of the system when working with complex plant extracts, affecting the reproducibility and yield of the synthesis [39,40]. Table 1 summarizes the reviewed articles on nanomaterials synthesized using extracts of *Annona*. They describe the extracts used, the materials of the nanomaterials, their characterization in terms of shape and size, and the biological activity evaluated.

## 4. Importance of Nanomaterials Synthesized with Plant Extracts of the Annona Genus

Nanoparticles exhibit interesting properties, including chemical stability, conductivity, and catalytic activity, which are related to biological activities such as antimicrobial, antifungal, antiviral, and anti-inflammatory effects [49,63,64,65]. Below, the activities and hypothesized mechanisms of *Annona* nanomaterials in biomedicine are described.

### 4.1. Antimicrobial Activity of Nanomaterials Synthesized with Annona Extracts

One of the most studied biological activities is antimicrobial activity, primarily due to the global issue of strains resistant to multiple antibiotics. One of the significant advantages of using nanomaterials is that bacterial strains cannot develop transmissible genetic resistance to these materials [3].

Two general hypotheses have been proposed regarding the mechanism by which antimicrobial activity occurs. The first mechanism involves damage to the cell membrane. The second mechanism predicts the development of oxidative stress, which in turn disrupts processes essential for cell survival, such as DNA replication, mRNA transcription, and protein translation from mRNA [1].

**Figure 3:**
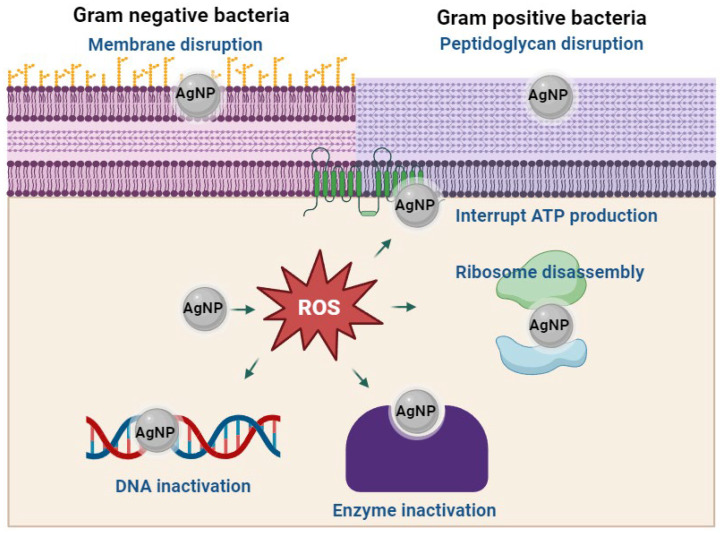
Mechanisms involved in the antimicrobial activity of AgNPs. At least two mechanisms have been proposed for the antimicrobial activity of nanomaterials. The first is direct interaction with the cell membrane, causing damage to its structure and disruption of the electron transport chain. This leads to a reduction in ATP production. The second mechanism involves the production of reactive oxygen species, which can disrupt essential cellular processes, including DNA replication, mRNA transcription, and protein translation from mRNA.

On the other hand, metal nanoparticles release small toxic ions that can pass through the pores of the membrane [1] or remain trapped within it, generating pores that increase its permeability, which in turn alters cellular transport, as well as induces a specific interaction. When the microbe possesses mitochondria, nanoparticles can affect the respiratory chain, suppress ATP production, and generate reactive oxygen species, leading to cell death [3,63]. It should be noted that antimicrobial activity depends on several factors, such as sensitivity (bacterial factors) and inherent characteristics of the nanoparticles (type, concentration). For example, nanomaterials, especially nanoparticles, which are smaller and less electronegative, interact more with the surface of microbial cell membranes, generating a strong electrostatic attraction and triggering antimicrobial activity [66]. Due to the extensive use of silver as a source of metal for nanoparticle production, the proposed mechanisms involved in the antimicrobial activity of silver nanoparticles are summarized in Figure 3.

**Figure 4:**
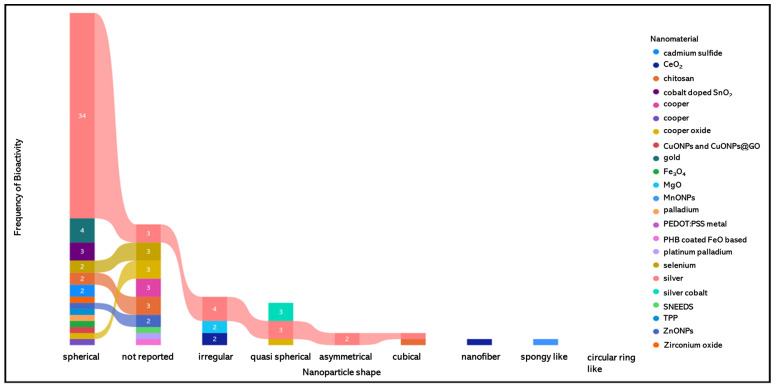
Global visualization of the distribution of nanoparticle morphologies synthesized using *Annona* extracts, as categorized by shape and associated nanomaterial type. The most frequently reported morphology is spherical (n = 34), followed distantly by irregular (n = 4), quasi-spherical (n = 3), and not-reported shapes (n = 13). Less common forms include asymmetrical, cubical, nanofiber, spongy-like, and circular ring-like structures. The figure also highlights that silver nanoparticles dominate across all shape categories, aligning with their prevalence in the literature. The Sankey-type flow diagram was generated by POWER BI, where colors represent distinct nanomaterials (e.g., Ag, CuO, ZnO, chitosan, etc.) and connections trace their association with specific nanoparticle shapes. This visualization highlights both the prevalence of silver-based systems and the diversity of biosynthesized structures derived from different *Annona* matrices.

Overall, phytosynthesis using *Annona* extracts yields a wide range of nanoparticle morphologies, compositions, and bioactivities. As shown in Figure 4, spherical nanoparticles are the most common shape, often derived from silver-based systems due to their ease of synthesis and superior antimicrobial properties. While this biosynthetic strategy presents notable ecological and biomedical advantages, it also reveals variability in nanoparticle properties across studies, reflecting the need for standardization in protocols and metabolite profiling. Nonetheless, the consistent bioactivity and adaptability of *Annona*-mediated nanomaterials position them as strong candidates for future development in green nanotechnology.

**Table 1:** Characterization and biological activity of nanomaterial Phytosynthesized using *Annona* extracts.

Specie	Tissue	Extract Solvent	Nanomaterial	Size (nm)	Shape	Bioactivity	Reference
*A. muricata*	Leaves	Methanol	CdS	3.42	Spherical	Antimicrobial activity against *Staphylococcus aureus* and antifungal activity against *Aspergillus niger*	[67]
*A. muricata*	Fruit	Water	CeO_2_	Not reported	Nanofiber	Antimicrobial activity against *Staphylococcus aureus* and *Enterococcus faecalis*	[68]
*A. reticulata*	Leaves	Water	CeO_2_	3.7–10.3	Irregular	Antioxidant and antidiabetic activity	[69]
*A. muricata*	Leaves	Chloroform	Chitosan	248–317	Not reported	Anticancer activity against HeLa cells	[62]
*A. muricata*	Leaves	Ethanol	Chitosan	234	Spherical	Antibacterial activity against *Escherichia coli* and *Salmonella typhimurium*	[63]
*A. muricata*	Leaves	Ethanol	Chitosan	282.75	Not reported	Not reported	[70]
*A. squamosa*	Leaves	Ethanol	Chitosan	535.1	Spherical	Cytotoxic against HeLa cells by induction of caspase-3 expression	[71]
*A. squamosa*	Leaves	Ethanol	Chitosan	531.1	Cubical	Induces caspase-3 expression on HeLa cells	[72]
*A. squamosa*	Leaves	Ethanol	Chitosan	535.1	Not reported	Induces caspase-3 expression and apoptosis	[73]
*A. squamosa*	Leaves	Ethanol	Chitosan	535.1	Not reported	Induces caspase-3 expression on WiDr cells	[72]
*A. muricata*	Leaves	Water	Cobalt-doped SnO_2_	0.33	Spherical	Antibacterial activity against *Pseudomonas aeruginosa* and *Staphylococcus aureus*, antifungal activity against *Candida albicans* and *Aspergillus niger*, antioxidant activity	[73]
*A. squamosa*	Stem barks	Water	Cu	Not reported	Not reported	Antimicrobial against *Staphylococcus aureus* and *Escherichia coli*, antifungal against *Candida albicans*, cytotoxicity against breast cancer MCF-7	[74]
*A. squamosa*	Seed	Water	Cu	5.99–24.48	Spherical	Insecticidal activity of *Anopheles stephensi* and *Tenebrio molitor* larvae	[75]
*A. muricata*	Fruit	Water	CuO	Not reported	Not reported	Antiproliferative activity against AMJ-13, MCF-7 breast cancer cell lines, and the human breast epithelial cell line (HBL-100)	[76]
*A. reticulata*	leaves	Water	CuO	Not reported	Not reported	Antioxidant and catalytic activity	[77]
*A. squamosa*	Seed	Water	CuO	11	Spherical	Antimicrobial against *Xanthomonas oryzae*	[78]
*A. squamosa*	Seeds	Ethanol	CuO	30.27	Semiglobular	Molluscicidal activity	[79]
*A. muricata*	Leaves	Water	CuONPs and CuONPs@GO	40	Spherical	Antibacterial activity towards both *Staphylococcus aureus* and *Salmonella typhi*	[80]
*A. muricata*	Leaves	Water	Au	25.55	Spherical	Antimicrobial against *Staphylococcus aureus*, *Enterococcus faecalis*, *Klebsiella pneumoniae*, and *Clostridium sporogenes*, antifungal against *Aspergillus flavus*, *Candida albicans*, *Fusarium oxysporum*, and *Penicillium camemeri*.	[81]
*A. muricata*	Peel and pulp	Water	Au	15	Spherical	Anticancer activity in treated Hep2 liver cancer cell line and non-toxic effect on regular VERO cell line	[82]
*A. muricata*	leaves	Ethanol 80%	Au	89.34	Smooth Spherical	Anticancer activity against metastatic melanoma MM-138 and primary melanoma FM-55, as well as breast cancer cell lines	[83]
*A. muricata*	leaves	Water	Fe_3_O_4_	23	Spherical	Antidiabetic activity	[84]
*A. squamosa*	Seeds	Water	MgO	27 to 68	Irregular	Antibacterial activity against *Pectobacterium carotovorum*, antioxidant activity and cytotoxicity against HeLa cells	[85]
*A. muricata*	Seed	Water	MnO	Not reported	Spongy-like agglomeration of smooth particles	Antimicrobial activity against *Escherichia coli* and *Staphylococcus aureus*	[86]
*A. squamosa*	Seed	Methanol	Pd	Less than 300	Spherical	Oxidative damage in hepatic tissue	[87]
*A. muricata*	Leaves	Not reported	PHB-coated Fe_3_O_4_–based	30 to 40	Not reported	Antiproliferative against HeLa and MDA-MB-231 cell lines	[88]
*A. muricata*	Leaves	Water	PtPd	3.97–10.68	Not reported	Antibacterial activity against *Escherichia coli* and *Staphylococcus aureus*	[89]
*A. reticulata*	Leaves	Water	Poly (3,4-ethylenedioxythiophene)	23.7	Circular ring-like	Not reported	[90]
			Poly (4-styrene sulfonate) Gold				
*A. muricata*	Fruit	Water	Se	Not reported	Not reported	Anticancer against lung cells (A-549)	[91]
*A. muricata*	Fruit	Water	Se	Not reported	Not reported	Antioxidant	[92]
*A. muricata*	Fruits	Water	Se	Not reported	Not reported	Antifungal against *Candida albicans*	[93]
*A. muricata*	Fruit	Water	Se	80–120	Spherical	Antioxidant, antimicrobial activity against *Escherichia coli*, *Pseudomonas aeruginosa*, *Klebsiella pneumoniae*, *Staphylococcus aureus*, *Enterococcus faecalis*, and *Listeria monocytogenes*	[94]
*A. diversifolia*	Leaves	Water	Ag	45 to 58	Spherical	Antibacterial activity against *Klebsiella pneumoniae* and *Enterobacter aerogenes*	[95]
*A. glabra*	Leaves	Water	Ag	10–100	Spherical	Larvicidal against *Aedes aegypti* and *Aedes albopictus* mosquito larvae	[60]
*A. glabra*	Fruit	Ethanol	Ag	7.11	Spherical	Antibacterial activity against *Pseudomonas aeruginosa* and *Escherichia coli*	[96]
*A. muricata*	Fruit Juice	Water	Ag	31.95	Spherical	Anticancer activity against HeLa cells, cytotoxicity against AMJ−13	[97]
*A. muricata*	Leaves	Water	Ag	10.87	Asymmetrical	Antimicrobial activity against *Escherichia coli*, *Staphylococcus aureus*, and *Enterococcus faecalis*, cytotoxicity against oral fibroblasts	[98]
*A. muricata*	Pulp	Water	Ag	51.5	Spherical	Antimicrobial activity against *Staphylococcus aureus*, *Enterococcus faecalis*, *Escherichia coli*, *Klebsiella pneumoniae*, *Proteus mirabilis*, and *Pseudomonas aeruginosa*, fungistatic action against *Candida albicans*	[61]
*A. muricata*	Pulp	Water	Ag	87	Spherical	Antimicrobial activity against *Staphylococcus aureus*, *Enterococcus faecalis*, *Escherichia coli*, *Klebsiella pneumoniae*, and *Proteus mirabilis*	[99]
*A. muricata*	Seeds	Water	Ag	62	Spherical	Antimicrobial activity against *Staphylococcus aureus*, *Enterococcus faecalis*, *Escherichia coli*, *Klebsiella pneumoniae*, and *Proteus mirabilis*	[99]
*A. muricata*	Seeds	Water	Ag	194	Spherical	Antimicrobial activity against *Staphylococcus aureus*, *Enterococcus faecalis*, *Escherichia coli*, *Klebsiella pneumoniae*, and *Proteus mirabilis*	[99]
*A. muricata*	Leaves	Water	Ag	205	Spherical	Antimicrobial activity against *Staphylococcus aureus*, *Enterococcus faecalis*, *Escherichia coli*, *Klebsiella pneumoniae*, and *Proteus mirabilis*	[99]
*A. muricata*	Leaves	Water	Ag	60	Spherical	Antimicrobial activity against *Pseudomonas aeruginosa* and fungistatic action against *Candida albicans*	[99]
*A. muricata*	Root bark	Water	Ag	22	Spherical	Antimicrobial activity against *Bacillus subtilis*, *Staphylococcus aureus*, *Klebsiella pneumoniae*, *Escherichia coli*, and *Pseudomonas aeruginosa*	[99]
*A. muricata*	Leaves	Water	Ag	Not reported	Not reported	Ability to cleave DNA into fragments	[100]
*A. muricata*	Leaves	Water	Ag	35	Spherical	Antimicrobial activity against *Klebsiella pneumoniae*, *Escherichia coli*, *Proteus vulgaris*, and *Staphylococcus aureus*	[101]
*A. muricata*	Leaves	Water	Ag	35	Spherical	Larvicidal activity against larvae of *Aedes aegypti*, *Anopheles stephensi*, and *Culex quinquefasciatus*	[102]
*A. muricata*	Peels	Water	Ag	11 to 23	Spherical	Antiproliferative against THP−1, HBL, and AMJ−13	[103]
*A. muricata*	Leaves	Water	Ag	35	Spherical	Antioxidant, antidiabetic, cytotoxic (HaCaT), and antimicrobial (*Staphylococcus aureus*, *Serratia marcescens*, and *Pseudomonas aeruginosa*)	[67]
*A. muricata*	Leaves	Ethanol	Ag	60.12	Spherical	Anticancer activity via CASP9 activation	[104,105]
*A. muricata*	Fruits	Ethanol	Ag	60.12	Spherical	Anticancer activity via CASP9 activation	[104,105]
*A. muricata*	Root	Water	Ag	34	Spherical	Antioxidant activity, selective cytotoxicity against HCT116, without affecting the growth of normal human lymphocytes and erythrocytes, and an anticancer agent for colon cancer	[106]
*A. muricata*	Leaves	Water	Ag	30 to 40	Not reported	Antimicrobial against *Staphylococcus aureus*, *Escherichia coli*, *Bacillus subtilis*, and *Pseudomonas aeruginosa*, antifungal activity against *Candida albicans*	[107]
*A. muricata*	Peel	Water	Ag	19.63	Quasi-Spherical	Antiproliferative against breast cancer (MCF−7, MDA-MB−468), colon cancer (HCT−116), and melanoma (A−375)	[108]
*A. reticulata*	Leaves	Water and ethanol	Ag	22	Not reported		[109]
*A. squamosa*	Leaves	Water	Ag	84.9	Irregular	Antimicrobial activity against *Escherichia coli*, *Bacillus subtilis*, *Xanthomonas campestris*, and *Staphylococcus aureus*, antifungal against *Aspergillus niger*	[110]
*A. squamosa*	Leaves	Different fractions	Ag	100–200	Cubical	Larvicidal activity against *Anopheles stephensi*	[111]
*A. squamosa*	Peel	Water	Ag	18–35	Spherical	Antioxidant activity examined by DPPH-scavenging assay and amylase inhibition,	[112]
*A. squamosa*	Leaves	Water	Ag	20–100	Spherical	Cytotoxic against MCF−7	[113]
*A. squamosa*	Leaves	Water	Ag	28.47	Spherical	Antimicrobial activity against *Bacillus cereus*, *Bacillus subtilis*, *Staphylococcus aureus*, *Salmonella typhimurium*, *Pseudomonas aeruginosa* and *Proteus vulgaris*	[114]
*A. squamosa*	Fruit	Water	Ag	15–50	Spherical	Antimicrobial activity against *Escherichia coli* and *Pseudomonas aeruginosa*	[115]
*A. squamosa*	Leaves	Water	Ag	35–90	Spherical	Antimicrobial activity against *Escherichia coli* and *Pseudomonas aeruginosa*	[116]
*A. squamosa*	Fruit	Ethanol	Ag	6.63	Spherical	Antibacterial activity against *Pseudomonas aeruginosa* and *Escherichia coli*	[96]
*A. squamosa*	Seed	Water	Ag	73.5	Irregular	Antimicrobial activity against *Escherichia coli*, *Bacillus subtilis*, *Xanthomonas campestris*, and *Staphylococcus aureus*, antifungal against *Aspergillus niger*	[110]
*A. squamosa*	Seeds	Water	Ag	22	Spherical	Larvicidal activity against mosquito *Anopheles stephensi* larvae	[116]
*A. squamosa*	Seeds	Water	Ag	50–80	Quasi-Spherical	Antimicrobial *Escherichia coli*, *Streptococcus mutans*, and *Staphylococcus aureus*	[117]
*A. squamosa*	Leaves	Water	Ag	52	Spherical	Antimicrobial against *Escherichia coli*	[96]
*A. muricata*	Leaves	Water	Ag	16.56	Quasi-Spherical	Antiproliferative against breast cancer (MCF−7, MDA-MB−468), colon cancer (HCT−116), and melanoma (A−375)	[108]
*A. muricata*	Leaves	Water	Ag-Co	39.34	pseudo-Spherically	Toxicity against *Drosophila melanogaster*, antibacterial against *Klebsiella sp., Salmonella* sp., *Streptococcus pneumoniae*, *Staphylococcus aureus*, and *Escherichia coli*, antifungal against *Candida albicans*	[81]
*A. muricata*	Leaves	Chloroform	SNEEDS	411.4	Not reported	Antioxidant	[62]
*A. muricata*	Leaves	Ethanol	TPP	234	Spherical	Antibacterial activity against *Escherichia coli* and *Salmonella typhimurium*	[63]
*A. muricata*	Fruit	Water	ZnO	29	Not reported	Antibacterial activity against *Escherichia coli*, *Klebsiella pneumoniae*, *Pseudomonas aeruginosa*, and *Staphylococcus aureus*, cytotoxicity against HCT116, K562.,	[118]
*A. muricata*	Leaves	Water	ZnO	80	Spherical	Anticancer against A549 and MOLT4	[20]
*A. reticulata*	Leaves	Water	ZrO_2_	13–20	Spherical	Antibacterial action against *Salmonella enterica* (multidrug-resistant)	[119]

Nanoparticles with antimicrobial activity against *Streptococcus mutans*, *Staphylococcus aureus*, and *Escherichia coli* strains have been synthesized from *A. squamosa* seed and leaf extracts [108,117]. The presence of phenolic compounds in the extract, combined with the natural antimicrobial effect of AgNPs, generates a synergistic effect on antimicrobial activity, resulting in a larger zone of inhibition for AgNPs synthesized with *Annona* compared to chemically synthesized AgNPs [66].

It is essential to acknowledge that the synthesis method of nanoparticles can also impact their biological activity. Assunção et al. [61] observed that exposure to artificial light during the synthesis of nanoparticles using pulp, seed, and leaf extracts of *A. muricata* generated AgNPs more rapidly and with greater antimicrobial and antifungal effects compared to the extracts and the AgNPs synthesized in darkness. Additionally, Jagtap et al. [114] demonstrated that the synthesis of AgNPs using *A. squamosa* leaf extract was faster and more efficient when microwaves were employed. Furthermore, they identified, using FT-IR (Fourier-transform infrared spectroscopy), that the biosynthesized AgNPs contained phenolic compounds and proteins that contributed to their stabilization. In addition to antimicrobial activity, the AgNPs also exhibited antioxidant and antidiabetic activity, as demonstrated by the α-amylase activity assay.

Some silver nanoparticles synthesized from extracts of *Annona*, such as *A. muricata* [98,120], *A. squamosa* [114], and *A. diversifolia* [95], have been tested against multidrug-resistant bacterial strains. Significant antimicrobial activity has been observed in addition to a reduction in the production of free radicals and skin inflammation caused by bacterial infection [115].

**Figure 5:**
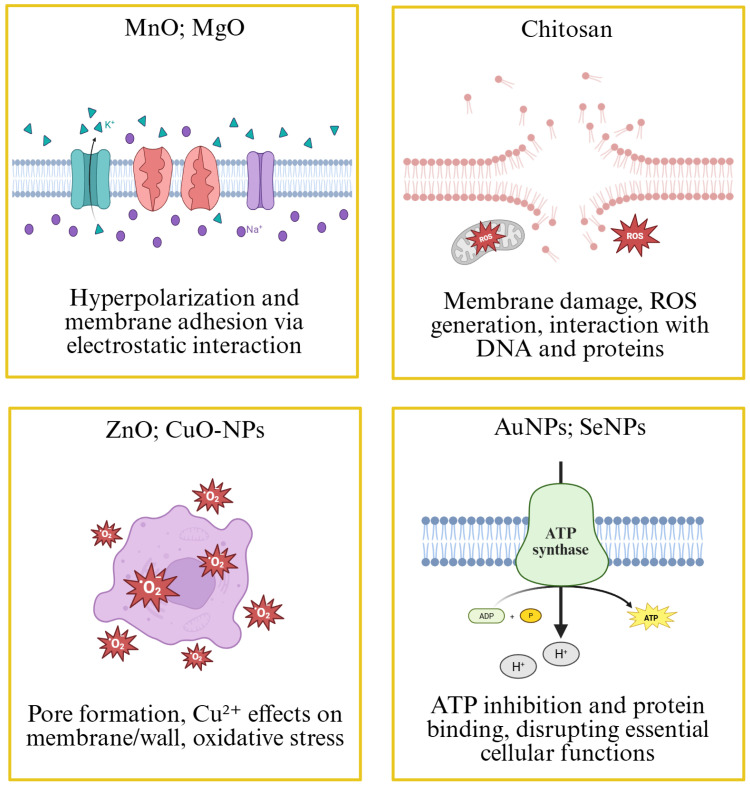
Comparative analysis of the antimicrobial mechanisms reported for different nanomaterials. This figure illustrates distinct modes of action exhibited by various nanomaterials. MnO and MgO act through membrane hyperpolarization and electrostatic adhesion. Chitosan induces membrane damage, generates reactive oxygen species (ROS), and interacts with DNA and proteins. ZnO and CuO nanoparticles disrupt microbial cells through pore formation, oxidative stress, and interactions of the metal ions (Cu^2+^) with the cell wall. AuNPs and SeNPs inhibit ATP synthase and bind to proteins, impairing essential cellular processes. These mechanisms reflect the diverse strategies employed by nanomaterials to exert antimicrobial and cytotoxic effects.

Meanwhile, other nanoparticles have been synthesized using extracts of the *Annona* genus. For example, the antimicrobial and antifungal activities of AuNPs synthesized with *A. muricata* leaf extract have also been demonstrated, with the activity increasing with the concentration of the AuNPs [81]. On the other hand, copper nanoparticles (CuNPs) have been synthesized using stem bark extracts of *A. squamosa*, exhibiting antibacterial activity against both Gram-positive (*Staphylococcus aureus*) and Gram-negative (*Escherichia coli)* strains, as well as antifungal activity against *Candida albicans* [79]. The antimicrobial activity of the CuONPs@GrapheneOxide nanocomposite was also compared with that of CuONPs, both of which were biosynthesized using *A. muricata* leaf extract, with CuONPs@GrapheneOxide being more efficient against *S. typhi* and *Staphylococcus aureus* [74,80]. Another material used for the preparation of nanoparticles with *A. muricata* extracts is chitosan. Nanoparticles synthesized from this material have demonstrated antimicrobial activity against strains such as *Escherichia coli* and *Salmonella typhimurium* [63]. Other used materials include zirconium oxide; nanoparticles synthesized with *A. reticulata* extract have shown antimicrobial activity against *Salmonella enterica* serotype Typhi, a multidrug-resistant strain [119]. Finally, metallic alloys have been tested to compare the efficacy of these bimetallic nanoparticles with that of nanoparticles made from a single material. Recently, the work of Akinsiku et al. [5] has been published, investigating the larvicidal, antimicrobial, and antifungal activities of bimetallic silver and cobalt nanoparticles. The results showed greater efficacy compared to non-alloyed AgNPs and CoNPs. Figure 5 summarizes the biological effects of nanoparticles sintered with various materials.

**Figure 6:**
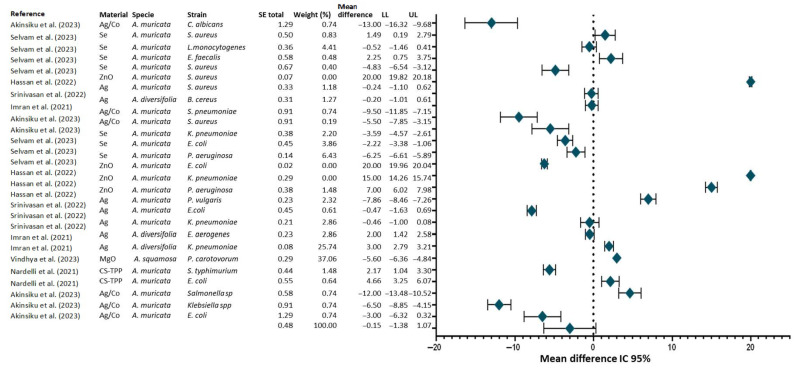
A forest plot of the inhibition of nanoparticles reported in this review. The graph shows the mean difference in inhibition halo size (in millimeters) for treatments based on nanoparticles synthesized with *Annona* extracts, compared to the respective controls included in each study. Each marker represents the mean difference for an individual study, and the horizontal lines indicate the 95% confidence interval (LL = lower limit; UL = upper limit). The vertical line at zero represents an absence of significant difference. Positive values indicate that the antimicrobial activity of the nanoparticle treatment is higher than that of the control, while negative values indicate that it is lower.

To perform a comparison of the efficiency as antimicrobials of nanomaterials synthesized with *Annona* extract, a forest diagram was generated (Figure 6). The highest concentration of points is recorded to the right of the vertical line at zero, suggesting that, in most studies, the nanoparticles increased the inhibition halo against various microbial strains. This finding indicates an improvement in the antimicrobial activity attributed to nanoparticles functionalized with plant extracts. In addition, some studies present 95% confidence intervals that do not cross the no-effect line (zero value), indicating that the observed differences are statistically significant in favor of the nanoparticles. In contrast, other studies present intervals that do cross the zero value, which prevents us from stating with certainty that there is a significant difference from the control in these cases. It is important to note that certain studies exhibit wide confidence intervals, suggesting a decrease in the precision of the effect estimate, which may be attributed to a limited number of replicates (most studies employ triplicates) or high variability in measurements. Overall, the results obtained support the hypothesis that nanoparticles synthesized from *Annona* extracts show superior antimicrobial activity to the control in multiple studies [63,74,95,118,121].

In addition, it can be observed that there is variability in the antimicrobial response depending on the type of strain. In general terms, greater inhibition was observed in Gram-negative strains, such as *E. coli*, compared to Gram-positive strains, such as *S. aureus*, as has been shown in previous research [63,95,118].

In summary, although Gram-negative bacteria commonly exhibit greater resistance to conventional antimicrobials due to their outer membrane, strains tend to show increased sensitivity to nanoparticles synthesized from *Annona* extracts. This hypothesis is based on the premise that the lipid structure of their outer membrane promotes the adhesion and penetration of nanoparticles, particularly when they are functionalized with bioactive compounds. The synergy between nanoparticles and plant extract metabolites could explain this greater efficacy against Gram-negative strains. It is worth noting that during the collection and analysis of the included studies, significant difficulties were encountered in identifying results that met the statistical requirements of quantitative meta-analysis. A considerable number of the articles examined did not provide complete information on primary data, such as the mean inhibition halos, dispersion measures (standard deviation or variance), and the sample size used. Given the above, it is concluded that, due to this circumstance, there is a need to take specific measures to address the situation effectively and efficiently.

### 4.2. Antiparasitic Activity

The antiparasitic activity of *Annona* species has been increasingly documented, with growing interest in their application against vectors of tropical and neglected, parasitic diseases. Extracts from various *Annona* plants, particularly *A. muricata*, *A. squamosa*, and *A. glabra*, are rich in bioactive compounds, including acetogenins, flavonoids, alkaloids, and terpenoids, which are known to interfere with essential biological pathways in parasites and insect vectors. These secondary metabolites are believed to exert their effects by disrupting mitochondrial respiration, interfering with membrane integrity, or generating oxidative stress in target organisms.

One of the most studied applications has been the larvicidal activity against disease-transmitting mosquitoes. For example, *Annona* extracts have demonstrated significant toxicity against the larvae of *Aedes aegypti* and *Aedes albopictus*, the primary vectors of Dengue, Zika, Chikungunya, and Japanese encephalitis, respectively [111,116]. In particular, silver nanoparticles (AgNPs) synthesized using *Annona glabra* extracts have shown enhanced larvicidal efficacy compared to the crude plant extract alone [84]. This improvement is attributed to the synergistic action of the metallic core and the capping phytochemicals, which together enhance nanoparticle stability, bioavailability, and toxicological effect on parasitic larvae.

Moreover, studies have reported that these phytosynthesized nanomaterials exhibit dose-dependent larvicidal activity with low toxicity to non-target organisms, suggesting their potential as eco-friendly alternatives to synthetic insecticides. This is especially important given the increasing resistance of mosquito populations to conventional chemical insecticides and the environmental and human health concerns associated with their prolonged use. Beyond vector control, some *Annona*-derived nanoparticles have also been investigated for their potential activity against protozoan parasites, such as *Leishmania* spp. and *Plasmodium* spp. [122,123,124]. However, further research is needed to establish their efficacy and safety profiles in vivo.

The antiparasitic potential of *Annona*-mediated nanomaterials not only contributes to sustainable and biocompatible strategies for vector management but also offers promising leads in the development of novel antiparasitic therapies with broad-spectrum activity and reduced environmental impact.

### 4.3. Other Activities

Other activities evaluated for nanomaterials synthesized with plants of the *Annona* genus include antioxidant and antidiabetic activities, as tested in CeO_2_ nanoparticles synthesized with *A. reticulata* leaf extract, which showed greater activity compared to chemically synthesized nanoparticles [68]. Likewise, the antioxidant and antidiabetic activity of AgNPs synthesized with *A. muricata* leaf extracts has been evaluated with positive results [105].

Although the use of *Annona* extracts in the synthesis of nanomaterials seems feasible. It must be recognized that the complexity of the extracts and the variability in their composition from extract to extract raise some doubts about the effectiveness of their use in the synthesis of nanoparticles, as the composition of phytochemicals varies within each plant and even within each tissue. It has been observed that the humidity, pH, and mineral composition of the soil, as well as the surrounding environment, significantly influence the phytochemical composition of plants. For this reason, strategies have recently been developed to control the concentration of specific compounds for the synthesis of nanomaterials. For example, Dalton et al. [125] synthesized gold nanoparticles coated with acetogenins and the protein transferrin, Peg-G-ACGs and Tf-Peg-G-ACGs, using the sonication method, obtaining particles with a size of less than 100 nm. They observed that these materials induce the mitochondrial permeability transition, which can optimize the transport system of drugs or bioactive molecules directly to cancer cells with greater efficiency.

### 4.4. Anticancer Activity

It is estimated that there will be 1,958,310 new cases of cancer and 609,820 cancer deaths in the United States this year [126]. Therefore, the search for new alternatives to current treatments is of utmost importance, especially in cases where the cancer is resistant to multiple drugs. Nanomaterials synthesized with plant extracts seem to be a viable option for this purpose. This is especially the case when plants intended for natural use contain phytochemical compounds or biomolecules that have previously been identified as anticancer agents, as is the case with plants of the *Annona* genus, which contain acetogenins and polyphenolic compounds known as “cancer killers” [20].

It has been proven that the efficiency of nanomaterials to produce a cytotoxic effect depends on the size, shape, and chemical surface of the nanomaterial; for example, it has been shown that nanoparticles with a diameter less than 100 nm can easily penetrate tumor cells through a retention effect and increased vascular permeation [94]. It has been suggested that the cytotoxicity triggered by nanoparticles in cancer cells can be produced by several mechanisms involving the generation of reactive oxygen species once they enter the cell, which in turn generate signals that trigger an apoptotic process. The apoptotic process produced by AgNPs is characterized by the presence of a contracted nucleus and loss of cell membrane integrity [94].

The activity of chitosan nanoparticles loaded with *A. squamosa* leaf extract has been evaluated in human colon cancer cells (WiDr), where it was observed that the expression of caspase 3 increased significantly, causing an arrest of the cell cycle in the G2/M transition, which in turn induced apoptosis in WiDr cells [72]; this phenomenon has also been observed in the lung cancer lineage (A549), both with SeNPs and ZnONPs [127,128], obtaining a greater antiproliferative effect compared to treatment with the extract. The AgNPs synthesized with aqueous extracts of *A. muricata* root produced alterations in the expression of genes related to the apoptotic process: PUMA; caspases 3, 8, and 9; Bax; and Bcl−2, as well as greater production of reactive oxygen species (ROS) in the colon cancer cell line (HCT116) [102,106]. The AgNPs synthesized from the aqueous extract of *A. muricata* peel induce impairment in autophagy in THP−1 monocytes through two mechanisms. The first is due to a reduction in the expression levels of IL−1, caspase 1, and ASC, which leads to cellular apoptosis via mitochondrial cell death. The second mechanism involves NLRP3, which is linked to lysosomal degradation processes [103].

CuNPs synthesized with the aqueous extract of the stem bark of A. squamosa do not exhibit significant antiproliferative activity in the breast cancer line MCF−7 [74]. In contrast, a significant selective cytotoxic effect of AgNPs and CuONPs has been characterized. NPs synthesized with leaf extracts of *A. squamosa* and *A. muricata*, respectively, when tested in the same cell line [76,113]. Similarly, a cytotoxic effect has been characterized in human keratinocyte cells (HaCaT) exposed to AgNPs synthesized with ethanolic extract of the fruit of *A. muricata* [120].

The use of a combination of two biosynthesized nanoparticles has been explored to achieve a synergistic effect in treatment. Mixing chitosan nanoparticles synthesized with *A. muricata* leaf extract and *Biancaea sappan* wood in a 1:8 ratio has a greater antiproliferative effect than when used individually [62]. In addition, researchers had encapsulated *Annona squamosa* seed oil using TPGS (d-α-tocopherol polyethylene glycol 1000 succinate) as a stabilizer, forming spherical nanoparticles smaller than 200 nm. They evaluated the antitumor potential of these nanoparticles. In vitro tests on 4T1 breast cancer cells demonstrated that the nanoparticles reduced the IC_50_ value by a factor of seven compared to the seed oil alone. In vivo experiments using mice with 4T1 tumors confirmed that the nanoparticle treatment (15 mg/kg) achieved the highest tumor inhibition rate (TIR) compared to plain seed oil. The study suggests that these nanoparticles could serve as effective carriers for delivering natural anticancer agents directly to tumor cells [35].

Despite growing interest in the green synthesis of nanoparticles using extracts from plants of the genus *Annona*, significant limitations remain in terms of evaluating their biocompatibility and toxicity. Although they contain bioactive metabolites with antioxidant, antimicrobial, and anticancer properties, their incorporation into nanoparticles can generate undesirable biological responses. A considerable amount of research focuses on physicochemical characterization and preliminary biological activity. However, there is a lack of systematic evaluations of their biosafety. Such evaluations include cytotoxicity assays in normal cells, genotoxicity studies, immunotoxicity assessments, and accumulation in organs. Recently [128] a published report explored blood compatibility in humans and the treatment of thrombosis using silver and gold nanoparticles synthesized from various biological resources, establishing that nanoparticle concentration is of utmost importance in the onset of hemolysis. Therefore, they suggest further studies for the implementation of silver and gold nanoparticles in the clinical treatment of thrombosis in humans.

Additionally, factors such as size, shape, dose, route of administration, and interactions with components of the biological environment can significantly modify the behavior and toxicity of nanoparticles within the body. The lack of standardization and limited toxicological information are significant obstacles to their clinical application, as the absence of robust data on their safety profile implies an inherent risk in progressing to preclinical or therapeutic trials.

## 5. Conclusions

In this review, a comprehensive analysis of 74 articles concerning the synthesis of nanomaterials employing extracts from the *Annona* genus was conducted. The analysis revealed a pronounced predilection for materials such as silver and gold over other materials. In addition to a documented predilection for leaves, there has been a notable surge in the publication of articles pertaining to antimicrobial activities and other in vitro trials.

Plants of the *Annona* genus are rich in phytochemicals that support the green synthesis of a wide variety of nanomaterials. However, more efficient extraction methods and optimized solvent systems are needed to maximize the use of bioactive compounds found in *Annona* tissues. For instance, acetogenins—potent but highly lipophilic molecules—are rarely utilized because they are poorly extracted with water, which remains the most common solvent in green synthesis protocols. Adopting organic or mixed-solvent systems could improve their recovery and incorporation into nanomaterials.

The current literature shows that *Annona muricata* is the most studied species, yet other species within the genus also exhibit rich phytochemical profiles, including alkaloids, phenolics, terpenes, and acetogenins, which warrant further exploration. Expanding research to less-studied *Annona* species could uncover new phytosynthetic capacities and broaden the diversity of nanomaterials obtained.

To improve reproducibility and scalability, there is a pressing need to standardize extraction and synthesis protocols. Critical parameters such as extract concentration, solvent polarity, pH, temperature, and reaction time must be consistently reported and optimized to ensure uniform nanoparticle properties and biological performance.

Future research should focus on more targeted applications, particularly for pathogens responsible for prevalent infectious diseases in tropical regions, where *Annona* species are widely available and culturally significant. In addition, promising nanomaterials with demonstrated in vitro bioactivity must be evaluated through in vivo studies to assess their true therapeutic potential, biodistribution, accumulation, signaling effects, and interactions with host molecules and organelles, which are factors that current in vitro models often fail to capture.

Addressing these challenges will be essential to unlock the full potential of *Annona*-derived nanomaterials and advance them toward safe, effective, and sustainable applications in biomedicine.
